# Completeness and Reliability of the Republic of South Africa National Tuberculosis (TB) Surveillance System

**DOI:** 10.1186/s12889-015-2117-3

**Published:** 2015-08-11

**Authors:** Laura Jean Podewils, Nonkqubela Bantubani, Claire Bristow, Liza E Bronner, Annatjie Peters, Alexander Pym, Lerole David Mametja

**Affiliations:** Division of Tuberculosis Elimination, U.S. Centers for Disease Control and Prevention, Atlanta, GA USA; Unit for Clinical and Biomedical Tuberculosis Research Unit, Medical Research Council of South Africa, Durban, South Africa; Global AIDS Program, U.S. Centers for Disease Control and Prevention, Pretoria, South Africa; Department of Tuberculosis Control, Republic of South Africa National Department of Health, Pretoria, South Africa; Global Tuberculosis Branch, Division of Global HIV/AIDS and Tuberculosis, Centers for Disease Control and Prevention, 1600 Clifton Road NE, MS E-10, Atlanta, GA 30333 USA

## Abstract

**Background:**

Accurate surveillance data are paramount to effective TB control. The Republic of South Africa’s National TB Control Program (NTP) has conducted TB surveillance since 1995 and adopted the Electronic TB Register (ETR) in 2005. This evaluation aimed to determine the completeness and reliability of data in the Republic of South Africa’s TB Surveillance System.

**Methods:**

Three of nine provinces, three subdistricts per province, and 54 health facilities were selected by stratified random sampling. At each facility, 30 (or all if <30) patients diagnosed in Quarter 1 2009 were randomly selected for review. Patient information was evaluated across two paper and four electronic sources. Completeness of program indicators between paper and electronic sources was compared with chi-square tests. The kappa statistic was used to evaluate agreement of values.

**Results:**

Over one-third (33.7 %) of all persons with presumptive TB recorded as smear positive in the TB Suspect Register did not have any records documenting notification, treatment, or management for TB disease. Of 1339 persons with a record as a TB patient at the facility, 1077 (80 %) were recorded in all data sources. Over 98 % of records contained complete age and sex data. Completeness varied for HIV status (53-86 %; *p* < 0.001) and DOT during the intensive phase of treatment (17-54 %; *p* < 0.001). Agreement for sex was excellent across sources (kappa 0.94); moderate for patient type (0.78), treatment regimen (0.79), treatment outcome (0.71); and poor for HIV status (0.33).

**Conclusions:**

The current evaluation revealed that one-third of persons diagnosed with TB disease may not have been notified of their disease or initiated on treatment (‘initial defaulters’). The ETR is not capturing all TB patients. Further, among patients with a TB record, completeness and reliability of information in the TB Surveillance System is inconsistent across data sources. Actions are urgently needed to ensure that all diagnosed patients are treated and managed and improve the integrity of surveillance information.

## Background

Accurate surveillance data are crucial to plan, implement, and evaluate TB control programs. South Africa is ranked by the World Health Organization (WHO) as the 6th highest among the top 22 high-burden countries for TB in the world, with an estimated 329,000 persons diagnosed with TB each year (incidence rate 860/100,000) [1]. South Africa’s National TB Control Program (NTP) has been monitoring TB case rates and treatment outcomes since 1995 [2]. In 2005, the Electronic Tuberculosis Registry (ETR) was implemented nationally as a vehicle to monitor key indicators essential to understanding the tuberculosis burden and management in South Africa [3–5]. The ETR has approximately 400 users at the sub-district, district, provincial and national levels of the TB program and contains over one million patient records [6, 7]. As part of the strategy to integrate TB and HIV services [8], the ETR also captures basic information on HIV status and HIV-related treatment among TB patients.

Since the ETR was introduced, there has not been a systematic evaluation of the National TB Surveillance System. Periodic evaluations of surveillance systems are critical to ensure the data are accurate and reliable and to guide public health programs [9]. Previous studies in specific communities reported incongruence between diagnosed TB cases in laboratory records and TB cases reported in the surveillance system [10]. Limitations of the utility of the ETR for health facilities and the local TB program have also been reported [11].

A WHO-led review of the NTP in July 2009 emphasized the need to systematically evaluate the TB Surveillance System in South Africa [12]. Specifically, the reviewers noted variability in the completeness and quality of records; a backlog of data entry; and incomplete understanding of TB indicators among some staff responsible for recording and reporting TB data. The committee recommended a comprehensive validation of the TB Surveillance System.

This project aimed to systematically evaluate the completeness and reliability of the TB Surveillance System in South Africa.

## Methods

### Study design

A retrospective data audit was performed to evaluate the accuracy of the TB Surveillance System for identifying persons with TB disease; the completeness of information from different sources; and the reliability of data from different sources.

### Study Population and selection of sites

The sampling strategy was determined in consultation with the National TB Program. The Republic of South Africa has nine provinces, each divided into districts and subdistricts (or local governmental units (LGUs) in provinces with no subdistricts). Provinces were divided into tertiles of cure rate for 2008 in the National ETR surveillance database, and one province was selected randomly from each tertile [13]. Within each province, subdistricts (or LGUs) were categorized according to the cure rate as above. One subdistrict from each tertile of cure rate was randomly selected. A full listing of NTP facilities managing TB patients was obtained for each subdistrict, and facilities were categorized as rural or urban based on National Statistics and consultation with district TB program managers [14]. One urban and one rural district/Level 1 hospital, community health center (CHC), and primary health clinic were each selected at random in each subdistrict (6/subdistrict). If a subdistrict did not have a facility in a particular category, a facility was randomly selected from the remaining facility categories.

### National TB program surveillance system overview

**Fig. 1:**
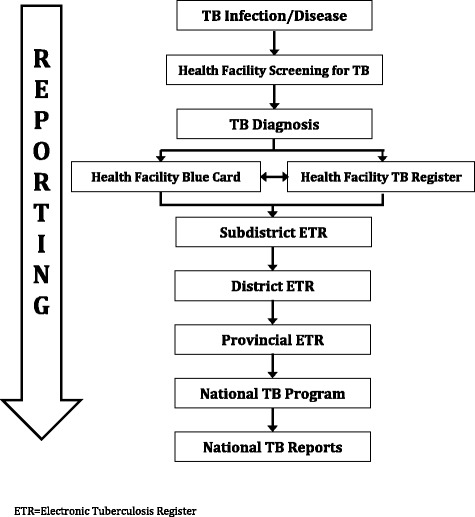
Schematic of the design of the TB Surveillance System for recording and reporting TB information for the National TB Program, South Africa

The National TB Surveillance System spans multiple levels of TB care and program management (Fig. 1). The NTP provides forms to health facilities for recording and reporting information on persons with presumptive TB and persons with TB disease, including the:

1. ***TB suspect register***, a logbook to record baseline information on persons with presumptive TB disease based on NTP Guidelines at the health facilities [2].Persons that are positive for the presence of acid-fast bacilli (AFB) on smear microscopy are subsequently recorded in the TB Register.
2. ***TB Blue Card***, the primary medical record for persons diagnosed with who initiate treatment.
3. ***TB Register***, a spreadsheet to record key TB information on all persons diagnosed with TB disease.

Separate from this paper-based system, the ***Electronic TB Register (ETR)*** is a software program the NTP uses to quantify, monitor, and evaluate TB burden and treatment outcomes. It is installed on computers at all subdistrict, district, provincial, and national offices.

Health facilities record information on person with presumptive TB in the TB suspect register. Specimens collected from persons with presumptive TB are sent to a centralized laboratory, where they are tested for TB using smear microscopy and microbacterial culture; a paper copy of the laboratory results are transmitted to the clinic via courier (the laboratory system is not directly linked with the surveillance system). A patient file, or TB Blue Card, is initiated for all persons diagnosed with TB disease, and key information is also recorded in the paper TB Register. The TB Registers are sent to the subdistrict (or local government unit) office, where the information is entered into the initial ETR. The database file from the ETR is transferred to the district TB program, where the data from all subdistricts within that district are merged. District ETR databases are provided to the provincial office, where again they are merged to represent the entire province. The provincial TB data is then sent to the NTP, where it is used to generate annual reports.

### Data audit and collection

#### Cross-check of persons with presumptive TB, TB Register, and TB patient management

To evaluate whether the TB Surveillance System is capturing persons with presumptive TB who are newly diagnosed with TB disease, the first 30(or all if <30) individuals documented as smear positive in the TB suspect register at each facility during Quarter 1 (Q1) 2009 were cross checked with TB Blue Cards and the paper TB Register.

Standardized forms were utilized to record: date of smear positivity, TB registration number, presence of TB Blue Card, patient listing in TB Register, and if the patient had died or was lost to follow up before initiating treatment. Only diagnostic smears were considered; date of sputum collection was cross-checked with date of initial presentation when the person was identified as having presumptive TB disease.

#### Data audit: TB patient records

For the data audit, 30 (or all if <30) TB Blue Cards were randomly selected from TB patients diagnosed in e Q1 2009. If Blue Cards were not available, patients were sampled from the TB Register.

Sociodemographic and clinical patient information was collected from each data source (TB Blue Card, TB Register, initial ETR, district ETR, provincial ETR, and national ETR) using standardized forms. Variables included: age, date of birth, patient category (new, retreatment after failure, retreatment after default, other retreatment, relapse), disease classification (pulmonary or extrapulmonary), pretreatment smear date and results, culture results, conversion smear date and results, HIV status, HIV treatments, whether or not the patient received DOT, treatment outcome date and treatment outcome.

### Statistical analysis

All analyses were conducted using Stata 13.0 (Galveston, Texas, USA). Frequencies and proportions were used to describe the facilities and patients included in the evaluation.

#### Proportion of TB cases identified and recorded

Overall counts were tallied to determine the number of total number of persons with presumptive TB documented in the suspect registers for Quarter 1 2009 at selected facilities, number with sputum results recorded, and number with a positive sputum smear for TB disease. The number of TB cases detected was divided by the total number of persons with presumptive TB in the suspect register with a recorded sputum smear result to calculate the proportion of cases among persons recorded as having presumptive TB and tested for TB disease. The proportion of persons with presumptive TB diagnosed with smear positive TB based on the TB suspect register that had a record in each the a) TB Blue Card file and b) TB Register was calculated.

#### Completeness

The total number of selected patients with a record in each data source was divided by the number of total number of persons with TB to yield a proportion for completeness of records for each source. Completeness for individual TB indicator variables was evaluated using the subset of TB patients with a record available in all data sources. For each variable of interest, the proportion of records with a non-missing value recorded was calculated, and the chi-square statistic was used to compare proportions across data sources.

#### Reliability

Using the subset of TB patients with a record available in all sources, reliability of the actual value recorded for key TB indicators was examined across data sources. The intraclass correlation coefficient was used to measure reliability for continuous variables (age) and the Cohen’s kappa coefficient was used to for categorical variables. Reliability for age was examined for all pairwise combinations. The kappa statistic was calculated for all pairwise comparisons; for 3-level comparisons: a) TB Blue Card, TB Registry, and initial ETR; b) TB registry, initial ETR, provincial ETR; c) initial ETR, provincial ETR, national ETR; and d) overall. For all comparisons at least one data source was required to have a non-missing value, such that credit of agreement was not granted when all sources were missing. An overall weighted kappa value was calculated to summarize overall reliability (weighted across all data sources) and account for the absence of subdistricts in some provinces.

### Ethical review

This project was reviewed and approved by the Ethics Committee of the South African Medical Research Council. The evaluation was also reviewed by the U.S. Centers for Disease Control and Prevention and determined to be non-research, thereby not requiring approval by the institutional review board for research in human subjects. The Republic of South Africa National Department of Health NTP and all provincial and district TB program offices provided written approvals prior to evaluation.

## Results

The three provinces selected for the study were Gauteng (GAU), KwaZulu-Natal (KZN), and Mpumalanga (MPU); eighteen facilities were selected from each province to yield a total of 54 facilities.

### Case detection

Using simple counts, a total of 8409 persons with presumptive TB were logged in the TB suspect registers at the selected facilities. Of these, 6853 (81.5 %) had a smear result recorded on the suspect register, with 857 (12.5 %) of those having a result recorded as a positive smear for TB (Table 1).

**Table 1:** Case finding from TB suspect registers in the 54 facilities sampled in the present evaluation, Quarter 1 2009

	KwaZulu-Natal n (%)	Mpumpulanga n (%)	Gauteng n (%)	Total n (%)
TB suspects	4042	1312	3055	8409
TB suspects with smear result recorded	2869 (71.0)	1091 (83.2)	2893 (94.7)	6853 (81.5)
TB suspects with positive sputum smear	380 (13.2)	205 (18.8)	272 (9.4)	857 (12.5)

Percentages (%) reflect proportion of the row immediately above

### Cross-check of TB suspects with the TB register and TB blue card

A total of 721 persons with presumptive TB recorded as smear positive in the TB Suspect Register (*i.e.*, diagnosed with and documented as having TB disease) were selected for more in-depth comparisons and analysis. Of these, 355 (49.2 %) had TB Blue Cards available and 457 (63.4 %) were documented in the paper TB Register at the facility (Table 2). Of 250 patients without a TB Blue Card and not recorded in the TB Register, 3 (1.2 %) were identified as lost to follow-up and 4 (1.6 %) patients were noted as having died before starting treatment. In total, over one-third (33.7 %) of all persons with presumptive TB diagnosed with TB and recorded as smear positive in the TB Suspect Register did not have any records documenting notification, treatment, or management for TB disease.

**Table 2:** Number and proportion of newly diagnosed smear positive TB cases in the suspect register with TB Blue Cards, entries in the TB Register, or noted as died or lost to follow-up prior to treatment initiation (n = 721)

		KwaZulu-Natal n (%)	Mpumpulanga n (%)	Gauteng n (%)	Total n (%)
	Total sample smear + TB cases from suspect register	294	174	253	721
	TB Blue Card	179 (60.9)	58 (33.3)	118 (46.6)	355 (49.2)
	TB Register	197 (67.0)	126 (72.4)	134 (53.0)	457 (63.4)
a	No Blue Card or Register	89 (30.3)	48 (27.6)	113 (44.7)	250 (34.7)
b	Died before treatment start^a^	2 (2.3)	1 (2.1)	1 (1.0)	4 (1.6)
c	Lost to follow-up before treatment start^a^	2 (2.3)	1 (2.1)	1 (1.0)	3 (1.2)
d	Number of smear + TB patients diagnosed and recorded on the suspect register with unknown status after laboratory results were received at the clinic; no notation of treatment or management	85 (28.9)	46 (26.4)	111 (43.9)	243 (33.7)

Died before treatment start and lost to follow-up before treatment start (“early defaulters”) as noted on the suspect register

^a^Percentages are of those without a TB Blue Card or in the TB Register

d = a - (b + c)

### Data audit of TB patient records

**Fig. 2:**
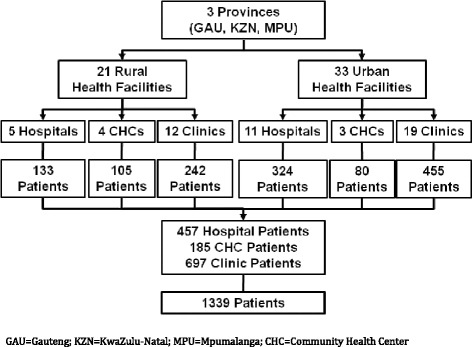
Overview of the study population of TB patients included in the data audit (n = 1339)

A total of 1339 persons with TB disease were selected for inclusion in the data audit of TB cases. Approximately one-third of the total records were reviewed from each province (Fig. 2). Records were almost equally distributed from rural and urban facilities in KZN and MPU, but most records in GAU were sampled from urban facilities. Over half (52.1 %) of all TB patients were diagnosed at clinics, one-third were sampled from hospitals (34.1 %), and under 15 % were from community health clinics (CHCs).

### Completeness

#### Patient records

Of 1339 patient records selected, 1273 (95.1 %) were recorded in the paper TB Register, 1222 (91.3 %) were recorded in the initial ETR, 1195 (93.1 %) were in the district ETR, 1185 (92.9 %) were in the provincial ETR, and 1199 (94.0 %) were in the national ETR. Of 1339 patients, 1077 (80.4 %) had records in all data sources.

#### Key TB indicators

Using only TB patients with records in all sources (n = 1077), data on age or date of birth, sex, and patient registration type was over 98 % complete across all data sources (Table 3). Information on key indicators was often more complete in the TB Register than on the TB Blue Card, notably for disease classification (pulmonary or extrapulmonary) (96.3 % TB Register vs. 78.6 % Blue Card), HIV status (85.8 % vs. 64.0 %), and treatment outcome (95.2 % vs. 79.8 %). Over half of the information on patient DOT during the intensive phase (53.7 % vs. 22.3 %) and over three-quarters of the information on DOT coverage at the end of treatment (20.4 % vs. 4.4 %) was lost between the paper TB Register and the initial ETR. Similarly, 31.9 % of the information documenting HIV status (85.8 % vs. 53.9 %) in the paper TB Register was not reflected in the ETR. Discrepancies were evident across electronic sources from the initial ETR to the national ETR, with the proportion of records with a non-missing value for a given variable generally declining with increasing levels of management. However, the national database had slightly more records with a value recorded for sex, treatment start and outcome dates, and treatment regimen than the initial ETR database.

**Table 3:** Completeness: number and proportion of patient records with a value recorded for select TB indicators, by data source (n = 1077)

Variable	TB Blue Card n (%)	TB Register n (%)	ETR Initial Database n (%)	ETR District Database n (%)	ETR Provincial Database n (%)	ETR National Database n (%)	p-value*
Age or date of birth	1071 (99.4)	1069 (99.3)	1072 (99.5)	1073 (99.6)	1073 (99.6)	1074 (99.7)	0.67
Sex	1063 (98.7)	1069 (99.3)	1069 (99.3)	1072 (99.5)	1074 (99.7)	1076 (99.9)	0.006
Patient category	1055 (98.0)	1061 (98.5)	1071 (99.4)	1073 (99.6)	1069 (99.3)	1071 (99.4)	0.0002
Disease classification	846 (78.6)	1037 (96.3)	1055 (98.0)	1054 (97.9)	890 (82.6)	885 (82.2)	<0.0001
Pretreatment smear date	666 (61.8)	694 (64.4)	627 (58.2)	649 (60.3)	568 (52.7)	568 (52.7)	<0.0001
Pretreatment smear result	634 (58.9)	675 (62.7)	622 (57.8)	696 (64.6)	563 (52.3)	561(52.1)	<0.0001
Culture result	132 (12.3)	117 (10.9)	45 (4.2)	47 (4.4)	44 (4.1)	43 (4.0)	<0.0001
Treatment start date	979 (90.9)	1053 (97.8)	1069 (99.3)	1074 (99.7)	1072 (99.5)	1075 (99.8)	<0.0001
Treatment regimen	991 (92.0)	1039 (96.5)	1075 (99.8)	1076 (99.9)	1071 (99.4)	1076 (99.9)	<0.0001
HIV status	689 (64.0)	924 (85.8)	581 (53.9)	---^a^	---^a^	---^a^	<0.0001
CD4 count	315 (29.2)	345 (32.0)	236 (21.9)	---^a^	---^a^	---^a^	<0.0001
On ARV at TB tx start	269 (25.0)	518 (48.1)	85 (8.0)	---^a^	---^a^	---^a^	<0.0001
On ARV (anytime dur tx)	264 (24.5)	474 (44.0)	172 (16.0)	---^a^	---^a^	---^a^	<0.0001
On CPT	354 (32.9)	538 (50.0)	257 (23.9)	---^a^	---^a^	---^a^	<0.0001
DOT, intensive phase	571 (53.0)	578 (53.7)	241 (22.3)	208 (19.3)	191 (17.7)	191 (17.7)	<0.0001
Smr conversion rslt, 2 mo	412 (38.3)	466 (43.2)	437 (40.6)	435 (40.4)	376 (34.9)	372 (34.6)	<0.0001
Smr conversion rslt, 3 mo	134 (12.4)	155 (14.4)	117 (10.9)	128 (11.9)	74 (6.9)	71 (6.6)	<0.0001
DOT, end of treatment	327 (30.3)	220 (20.4)	47 (4.4)	39 (3.6)	28 (2.6)	30 (2.8)	<0.0001
Treatment outcome date	857 (29.6)	1009 (93.7)	1043 (96.8)	1054 (97.9)	1051 (97.6)	1045 (97.0)	<0.0001
Treatment outcome	859 (79.8)	1025 (95.2)	1044 (96.9)	1042 (96.8)	1047 (97.2)	1044 (96.9)	<0.0001

Note: the initial and district database are equivalent for the KZN province. Therefore completeness was counted in both columns for KZN if there was a value recorded for the given variable in the initial ETR database

^*^Chi-squared comparison of proportions across all columns

^a^Information on HIV status is not available at a patient level at higher levels of administration for reasons of confidentiality. Completeness of the data for HIV status at these levels of the ETR was therefore not able to be determined

### Reliability

Data on patient age and sex demonstrated high consistency across all data sources evaluated, with pairwise intraclass correlation coefficients ranging from 0.93 to 1.0 for age, and an overall kappa value of 0.94 for sex (Tables 4a-l and 5). Information on patient category and disease classification varied slightly between paper sources and electronic files, but was moderately reliable (kappa range 0.57-0.99). Information on the initial smear result showed inconsistencies from the TB Blue Card to the TB Register (kappa 0.62), from the TB Register to the initial ETR (kappa 0.58) and across electronic databases (kappa range 0.55-0.96). Documentation of the initial treatment regimen varied slightly between the TB Register and the initial ETR (kappa 0.80), but had excellent agreement across the ETR sources (kappa range 0.94-0.99). Information on HIV status was only available in the TB Blue Card, TB Register, and initial ETR database: agreement was poor overall (0.33) and for all pairwise (kappa range 0.11-0.41) comparisons. There was poor consistency across sources for DOT coverage during the intensive treatment phase and at the end of treatment, and likewise for smear conversion results for new and retreatment patients. Information noting treatment outcome had moderate agreement between paper sources, largely due to outcome not being recorded on the Blue Card (kappa 0.66).

**Table 4:** a-o. Pairwise measures of agreement for select sociodemographic and TB indicator variables for patients with records in all data sources (n = 1077)

	TB Register	Initial ETR	District ETR	Provincial ETR	National ETR
**a) Age**					
**TB Blue Card**	0.99	0.98	0.93	0.95	0.95
**TB Register**		0.98	0.94	0.95	0.95
**Initial ETR**			0.96	0.97	0.97
**District ETR**				0.99	0.99
**Provincial ETR**					1.0
**National ETR**					
**b) Sex**					
**TB Blue Card**	0.93	0.91	0.89	0.90	0.90
**TB Register**		0.94	0.90	0.92	0.93
**Initial ETR**			0.95	0.95	0.95
**District ETR**				0.99	0.99
**Provincial ETR**					1.0
**National ETR**					
**c) Patient category (new, relapse, retreatment after failure, retreatment after default, other)**					
**TB Blue Card**	0.82	0.72	0.57	0.63	0.64
**TB Register**		0.81	0.64	0.71	0.72
**Initial ETR**			0.81	0.86	0.87
**District ETR**				0.94	0.94
**Provincial ETR**					0.99
**National ETR**					
**d) Disease classification (pulmonary or extrapulmonary)**					
**TB Blue Card**	0.81	0.85	0.71	0.81	0.80
**TB Register**		0.87	0.63	0.83	0.82
**Initial ETR**			0.91	0.95	0.95
**District ETR**				0.99	0.99
**Provincial ETR**					1.0
**National ETR**					
**e) Pretreatment smear result (negative, positive, contaminated)**					
**TB Blue Card**	0.62	0.46	0.33	0.30	0.30
**TB Register**		0.58	0.43	0.40	0.40
**Initial ETR**			0.70	0.55	0.55
**District ETR**				0.56	0.56
**Provincial ETR**					0.96
**National ETR**					
**f) Treatment regimen (2RHEZ/4RH, 2RHZES/1RHEZ/5RHE, RHZ/4RH, Other, INH)**					
**TB Blue Card**	0.72	0.69	0.68	0.66	0.67
**TB Register**		0.80	0.73	0.77	0.78
**Initial ETR**			0.94	0.95	0.96
**District ETR**				0.98	0.99
**Provincial ETR**					0.99
**National ETR**					
**g) HIV status (negative, positive, recorded unknown)**					
**TB Blue Card**	0.41	0.11			
**TB Register**		0.38			
**Initial ETR**					
**District ETR**					
**Provincial ETR**					
**National ETR**					
**h) DOT during intensive phase (no, yes)**					
**TB Blue Card**	−0.21	−0.20	−0.13	−0.13	−0.14
**TB Register**		−0.13	−0.14	−0.10	−0.10
**Initial ETR**			−0.10	−0.05	−0.07
**District ETR**				- 0.09	−0.11
**Provincial ETR**					−0.04
**National ETR**					
**i) Smear conversion result – 2 month (negative, positive, contaminated)**					
**TB Blue Card**	0.19	−0.01	−0.11	−0.15	−0.13
**TB Register**		0.11	−0.07	−0.02	−0.01
**Initial ETR**			0.11	0.08	0.09
**District ETR**				0.28	0.29
**Provincial ETR**					0.85
**National ETR**					
**j) Smear conversion result – 3 month (negative, positive, contaminated)**					
**TB Blue Card**	−0.23	−0.35	−0.42	−0.38	−0.37
**TB Register**		−0.25	−0.45	−0.27	−0.27
**Initial ETR**			−0.34	−0.29	−0.26
**District ETR**				0.02	0.01
**Provincial ETR**					0.78
**National ETR**					
**k) DOT, end of treatment (no, yes)**					
**TB Blue Card**	−0.52	−0.09	−0.03	−0.06	−0.06
**TB Register**		−0.19	−0.11	−0.11	−0.11
**Initial ETR**			−0.07	−0.08	−0.12
**District ETR**				−0.07	−0.07
**Provincial ETR**					−0.05
**National ETR**					
**l) Treatment outcome (cured, completed, defaulted, failure, died, transferred out, moved out)**					
**TB Blue Card**	0.66	0.55	0.48	0.50	0.50
**TB Register**		0.74	0.60	0.68	0.68
**Initial ETR**			0.85	0.87	0.87
**District ETR**				0.96	0.96
**Provincial ETR**					0.97
**National ETR**					

Values represent intraclass correlation coefficients for the continuous variable of age, and kappa statistics for categorical variables

Comparisons with district/2nd-level ETR only include Gauteng and Mpumulanga provinces, as the initial ETR in KwaZulu-Natal is at the district level, and the data is included as initial ETR for KwaZulu-Natal

**Table 5:** Multi-level kappa values for select sociodemographic and TB indicator variables for patients with records in all data sources (n = 1077)

	TB Blue Card vs. register vs. initial ETR	TB Register vs. initial ETR vs. provincial ETR	Initial ETR vs. provincial vs. national ETR	Overall weighted (5-level for KZN, 6-level for GAU, MPU)
Sex	0.93	0.94	0.97	0.94
Patient category	0.79	0.79	0.90	0.78
Disease classification	0.83	0.88	0.97	0.84
Pretreatment smear result	0.59	0.56	0.70	0.63
Treatment regimen	0.74	0.84	0.97	0.81
HIV status	0.33			
DOT, intensive phase	−0.09	0.01	0.32	0.17
Smear conversion result, 2 month	0.25	0.23	0.40	0.35
Smear conversion result, 3 month	−0.03	−0.08	0.11	0.09
DOT, end of treatment	−0.30	−0.28	0.24	−0.09
Treatment outcome	0.65	0.76	0.90	0.72

Overall comparison includes all 5 levels for KZN and 6 levels for GAU and MPU

Data on HIV and HIV-related treatment only available through the initial ETR level

### Observational findings

There were several observations of the surveillance system noted during the audit. Individual patients do not have a unique TB registration number, so there are multiple patients with the same number once the data is merged across facilities, subdistricts, and districts. In addition, the system is not networked, which inhibits the ability to track patients who transfer or move. Finally, the system is not directly linked to the laboratory or other relevant surveillance systems, such as HIV, and there are no consistent mechanisms in place to reconcile patient information across systems.

## Discussion

The current evaluation revealed that information in different components of the South African National TB Surveillance System is often incomplete and inconsistent.

Over one-third of patients documented as smear positive in the Suspect Register (*i.e.*, confirmed laboratory diagnosis were not registered in the paper TB Register and did not have a TB Blue Card on file at the facility. This absence of recording and reporting suggests there are a number of persons with presumptive TB identified as having TB disease who may not be aware of their disease status and who may not be receiving TB treatment (‘initial defaulters’). This represents a missed opportunity for TB control, as these are persons who already entered the public health system but were not followed up or managed. These individuals are likely to experience increased morbidity and continue to spread TB in the community.

Additionally, this study demonstrated that different data sources reflect different numbers of TB cases. Underestimating the true number of TB patients managed at facilities inhibits the local and NTP’s ability to properly allocate human and logistical resources necessary to manage and treat TB patients. Among patients with records in all data sources, sociodemographic data appears to be largely retained between paper and electronic files. However, almost half the initial ETR database records were missing values for HIV status, and over three quarters of records were missing information on patient DOT.

This evaluation identified that even when information is available in all sources, often the values differ. The pairwise analyses of patient category, disease classification, pretreatment smear result, treatment regimen, and treatment outcome data ranged from satisfactory to excellent which may be a testament to their programmatic relevance. However, pairwise and multi-level comparisons of information on DOT, two and three month smear results, and HIV related data demonstrate a failure of the surveillance system and demand action for resolution. The overall concordance of the data shows a troubling incongruence with the trend being such that the data across electronic sources (*i.e.*, the ETR system at all programmatic levels) are more consistent than when paper sources are included. This may be attributed to differences in data quality management of paper and electronic systems or may be due to algorithms and decision rules that are part of the ETR computer system programming.

**Fig. 3:**
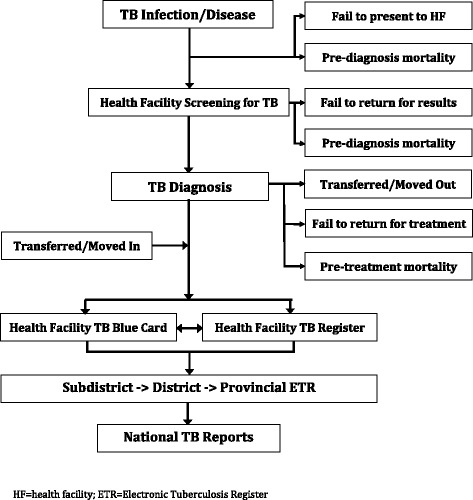
Schematic of the TB Surveillance System, including identified challenges that may compromise the completeness and reliability of recording and reporting TB disease, South Africa

The current evaluation also identified multiple challenges that may inhibit the linear flow and transfer of information as the TB Surveillance System is designed (Fig. 3). Though the NTP guidelines indicate all persons with presumptive TB who are diagnosed with TB are to be recorded in the TB Register, our findings revealed that these confirmed TB patients are often not recorded or reported. The system also lacks the capacity to track or reconcile information on patients who move or are transferred. Individual TB patients are not assigned a unique TB number; therefore it is not possible to link information if a patient is listed twice after seeking care at more than one facility. These observations challenge the NTP’s capacity to accurately monitor the TB burden and evaluate the management and outcomes of TB patients.

## Conclusions

These findings suggest that one third of persons diagnosed with TB are not started on treatment or notified of their disease. Further, the information for persons with a TB record who are being managed at a health facility will differ according to different levels of management and will have different implications for guiding program activities. Because the ETR is fully implemented, items of information about the same patient on the blue card, the TB register, and all levels of the ETR are expected to be identical and redundant. The ETR is expected to replace other sources of redundant data except for paper sources at the facility. The sources of the discrepancies identified in the current study are unclear; however, differences between paper sources and the initial ETR may be due to data entry errors or updates made to the ETR without revision or documentation in the paper register. Inconsistencies between levels of the ETR may also be due to information being updated at one level without ensuring other levels of the ETR are updated, or due to problems with the merging process. It is evident that a well-structured quality control and assurance process is needed to improve the reliability of the TB Surveillance System.

The information in the national ETR is the basis for evaluating, prioritizing needs, and allocating resources for the entire NTP and for generating annual statistics. Implementing measures to ensure all persons diagnosed with TB are properly retained and managed, and unifying paper, electronic, and laboratory systems may improve the integrity of the TB Surveillance System and also help to control and prevent the spread of TB in South Africa.
